# Mapping the Safety Profile of Histone Deacetylase Inhibitors in Children with Cancer

**DOI:** 10.1186/s13148-026-02192-z

**Published:** 2026-07-07

**Authors:** Alessandra Cianflone, Francesco Fabozzi, Fabiana Cacace, Emanuela Rossitti, Stefania Nappo, Francesca Cillo, Alessia Scarpa, Peppino Mirabelli, Francesco Paolo Tambaro

**Affiliations:** 1 Clinical and Translational Research Unit, AORN Santobono Pausilipon-IRCCS, Naples, Italy; 2 Department of Oncology, Hematology and Cellular Therapy, AORN Santobono Pausilipon-IRCCS, Naples, Italy; 3 Stem Cell Transplantation and Cell Therapy Unit, AORN Santobono Pausilipon-IRCCS, Naples, Italy; 4 Cell Processing and Immunogenetics Unit, Ba.S.C.O, AORN Santobono Pausilipon-IRCCS, Naples, Italy; 5 Dipartimento di Scienze Mediche Traslazionali, Sezione di Pediatria, Università degli Studi di Napoli Federico II, Napoli, Italia; 6 Department of Clinical Medicine and Surgery, Hematology Unit Federico II, University Medical School, Naples, Italy

**Keywords:** HDACi, Pediatric Cancer, Epigenetics, Treatment-related toxicity

## Abstract

**Background:**

Epigenetic alterations, including aberrant DNA hypermethylation and histone modifications, contribute to oncogenesis by disrupting normal gene expression programs. Unlike genetic mutations, these changes are potentially reversible, providing a strong biological rationale for the development of histone deacetylase inhibitors (HDACi) for therapy. Several HDACi are currently under investigation, either as monotherapy or in combination with other anticancer agents. A comprehensive understanding of toxicity is essential to appropriately balance risks and benefits, particularly because HDACi are most frequently evaluated within combination regimens. To date, no epigenetic agents have received regulatory approval for pediatric malignancies, and clinical development in this population remains at an early stage. The aim of this study was to systematically characterize the toxicity profiles associated with HDACi administration in pediatric patients with cancer, including both solid tumors and hematologic malignancies. To this end, we conducted a systematic review of the literature in accordance with the Preferred Reporting Items for Systematic Reviews and Meta-Analyses (PRISMA) guidelines.

**Results:**

Toxicity profiles were extracted from twelve studies investigating six HDACi: panobinostat, vorinostat, entinostat, pracinostat, valproic acid, and depsipeptide. Eleven studies were Phase I or Phase I/II clinical trials, and one was a retrospective study. Patients with solid central nervous system tumors represented the most frequently studied population. Overall, treatment-related toxicities were predominantly mild, with few severe (grade 4) adverse events, mainly within the hematologic category. According to the Common Terminology Criteria for Adverse Events grading system, hematologic toxicities were the most common adverse events across all HDACi, particularly thrombocytopenia, followed by mild gastrointestinal and metabolic toxicities. Cardiac, neurological, respiratory, and systemic toxicities were generally mild, less frequent, and considered treatment-related. Dose and route of administration influenced both the frequency and severity of toxicities. Pan-HDAC inhibitors were associated with the highest rates of toxicity.

**Conclusions:**

HDACi-related toxicities were moderate and manageable in pediatric patients. Differences in toxicity were observed among treatments, with higher doses linked to increased toxicity. These findings support further clinical trial development of HDACi in pediatric malignancies. However, Phase I design, small patient numbers, and heterogeneity in age, weight, and disease characteristics are major limitations in accurately assessing HDACi–induced toxicity.

**Supplementary Information:**

The online version contains supplementary material available at https://doi.org/10.1186/s13148-026-02192-z.

## Background

Epigenetic therapies have emerged as a promising class of targeted agents in children with cancer. Pediatric tumors, contrarily to adult cancers often exhibit a relatively low mutational burden but profound alterations in chromatin organization, transcriptional regulation, and developmental gene programs. These features render pediatric cancers particularly susceptible to therapeutic strategies targeting reversible epigenetic mechanisms, including DNA methylation, histone modifications, and chromatin remodeling [1–3]. Epigenetic modifications are heritable and reversible regulatory processes that influence gene expression without altering the underlying DNA sequence. Key epigenetic mechanisms play a fundamental role in regulating chromatin accessibility, DNA repair, and the maintenance of genomic stability, thereby influencing how genetic information is organized and expressed within the cell [4]. Dysregulation of these processes can impair DNA damage responses and promote genomic instability, thereby contributing to tumor initiation and progression [5–7]. Given their reversibility and regulatory breadth, epigenetic alterations represent attractive therapeutic targets. Among epigenetic compounds, HDACi are the most extensively investigated class in pediatric oncology. Histone acetylation is a key determinant of chromatin structure and transcriptional activity [8, 9]. Aberrant HDAC activity contributes to oncogenesis through multiple mechanisms, including silencing of tumor suppressor genes, promotion of uncontrolled proliferation, impaired differentiation, and resistance to apoptosis [10, 11]. HDACi induce hyperacetylation of histone and non-histone proteins, leading to transcriptional reprogramming, cell cycle arrest, apoptosis, and modulation of DNA repair and immune-related pathways. Several structurally and functionally distinct HDACi have entered preclinical and clinical evaluation in pediatric oncology, including hydroxamic acids, benzamides, cyclic peptides, and short-chain fatty acids. Despite sharing a common epigenetic target, these agents differ substantially in HDAC class selectivity, pharmacokinetics, and biological effects, factors that influence both efficacy and safety [12, 13]. In particular, they may be associated with a predictable, dose-dependent toxicity profile that primarily reflects their broad effects on transcriptional regulation [14, 15]. Importantly, given the fundamental role of epigenetic regulation in growth and development, the long-term safety of HDAC inhibition in children remains incompletely characterized. Potential late effects on neurodevelopment, endocrine function, and organ maturation underscore the need for careful toxicity monitoring and long-term follow-up in pediatric patients receiving epigenetic therapies. This systematic review aims to synthesize available preclinical and clinical evidence of HDACi in pediatric oncology, with a specific focus on toxicity profiles and safety considerations. By critically evaluating agent-specific and class-related adverse effects, this review aims to define the therapeutic limitations of HDAC inhibitors and support an informed risk-benefit assessment for their clinical development in pediatric oncology.

## Methods

The paper was reported in accordance with the Preferred Reporting Items for Systematic Reviews (PRISMA) guidelines [16]. All data originate from previously published papers in international peer-reviewed journals. The PRISMA checklist is shown in Supplementary Table 1. The review was not registered and no protocol was prepared.

### Outcome Measures

The primary outcome of this study was to evaluate the toxicity profile associated with HDACi treatment in pediatric oncologic diseases, encompassing both solid and hematologic malignancies. Toxicity was assessed through a qualitative analysis of reported adverse events, classified by toxicity type and severity grade (grades 1–5), as well as the occurrence of dose-limiting toxicities (DLTs).

### Literature Search

Relevant studies were identified through electronic searches in PubMed, Embase, Web of Science and clinicaltrial.gov for eligible studies published from 2000 to 2025. Studies conducted before the year 2000 were excluded, as HDACi were not available for clinical research purposes prior to this date. The last search was performed on 13 September 2025. The search strategy was conducted by the combination of the following terms for titles and abstracts: (“histone deacetylase inhibitor " OR " HDAC " OR " HDACi " OR " vorinostat " OR " romidepsin " OR " panobinostat” OR " belinostat " OR " valproic acid " OR " valproate " OR " entinostat " OR " tacedinaline " OR " domatinostat " OR " mocetinostat " OR " rocilinostat " OR " chidamide " OR " trichostatin A " OR " TSA " OR " givinostat " OR " abexinostat " OR “quisinostat” OR “pracinostat” OR “nanatinostat” OR “resminostat” OR “pyroxamide” OR “fimepinostat” OR “tefinostat” OR “AR-42” OR “pivanex” OR “nicotinammide” OR “sirtinol” OR “cambinol” OR “selisistat”) AND (leukemia OR leukemi* OR leukaemi* OR “childhood ALL” OR AML OR lymphoma OR lymphom* OR hodgkin OR hodgkin* OR T-cell OR B-cell OR non-hodgkin OR sarcoma OR sarcom* OR sarcoma, Ewing’s OR Ewing* OR osteosarcoma OR osteosarcom* OR “wilms tumor” OR wilms* OR nephroblastom* OR neuroblastoma OR neuroblastom* OR rhabdomyosarcoma OR rhabdomyosarcom* OR teratoma OR teratom* OR hepatoma OR hepatom* OR hepatoblastoma OR hepatoblastom* OR PNET OR medulloblastoma OR medulloblastom* OR PNET* OR “neuroectodermal tumor” OR retinoblastoma OR retinoblastom* OR meningioma OR meningiom* OR glioma OR gliom* OR “pediatric oncology” OR “paediatric oncology” OR “childhood cancer” OR “childhood tumor” OR “childhood tumors” OR “brain tumor*” OR “brain tumour*” OR “brain neoplasms” OR “central nervous system neoplasm” OR “central nervous system neoplasms” OR “central nervous system tumor*” OR “central nervous system tumour*” OR “brain cancer*” OR “brain neoplasm*” OR “intracranial neoplasm*”) AND (“toxicity” OR “toxicities” OR “safety” OR “tolerability” OR “adverse effect” OR “adverse effects” OR “side effect” OR “side effects” OR “toxic” OR “adverse drug reaction” OR “drug related side effects” OR “adverse reactions” OR “cytotoxicity” OR “cytotoxicities” OR “drug-induced toxicity” OR “treatment-related toxicity” OR “complications” OR “complication”). The search was limited to English-language articles.

### Study Selection

We selected studies evaluating HDACi administered as single-agent therapy in pediatric tumors. Three reviewers (FC, FF, PM) independently reviewed the search strategy. Titles and abstracts of studies were first evaluated independently by four authors of this paper (AS, ER, FC, SN). The authors independently reviewed the selected published articles. Any disagreements that arose between the reviewers were resolved through discussion with other two authors (FPT, AC). If the abstracts fulfilled the inclusion criteria, the full text was reviewed and data extracted.

### Eligibility Criteria and Data Abstraction

The inclusion criteria were as follows: Clinical Study, Clinical Trial, Clinical Trial, Phase I, Clinical Trial, Phase II, Clinical Trial, Phase III, Clinical Trial, Phase IV, Comparative Study, Controlled Clinical Trial, Equivalence Trial, Evaluation Study, Multicenter Study, Observational Study, Adaptive Clinical Trial, Randomized Controlled Trial, Validation Study. Studies including individual case reports, letters, reviews, studies reporting other diseases than pediatric tumors, clinical trials with no results, studies investigating the co-administration of epigenetic compounds in combination with other therapies, preclinical studies, in vitro studies, and nonhuman research were excluded. Studies were included when HDACi-related toxicity could be extracted or attributed according to the source report; combination studies without attributable toxicity data were excluded. The following characteristics were extracted: the first author’s name, publication time, study classification, clinical trial ID, disease type, disease stage, sample size, age, sex (male), study design, HDACi treatment and dose, any HDACi treatment interruption, adverse effects (AEs), DLTs, maximum tolerated dose/ recommended phase 2 dose (MTD/RP2D), any other therapies, clinical outcome measures. Only the toxicity data related to the MTD/RP2D were extracted. When described in aggregated form, they were analyzed separately.

### Risk of Bias Assessment

The methodological quality of the non-randomized studies included in this review was assessed using the Methodological Index for Non-Randomized Studies (MINORS), following the criteria originally described by Slim et al. [17].

### Data Analysis

All data were systematically analyzed using descriptive statistical methods to summarize study characteristics and outcomes. The data were expressed as the percentage of patients who experienced the adverse event. The overall frequencies of grade 1–5 events were reported for each study.

## Results

### Search Results

**Figure 1:**
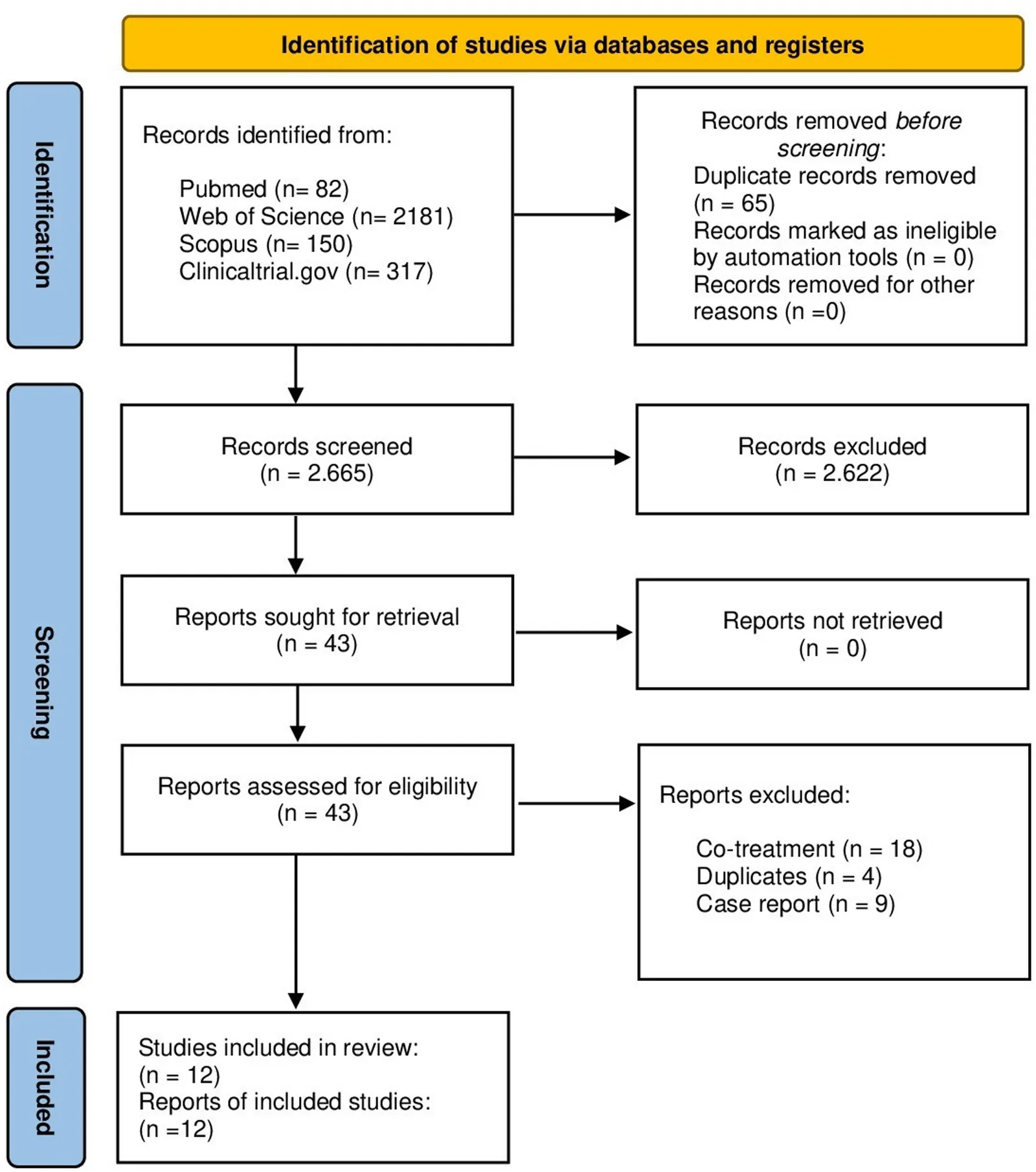
PRISMA flow diagram of the selected studies.

After the primary search, 2,730 titles of studies were evaluated. After removing duplicates (*n* = 65), the titles of the remaining 2,665 studies were evaluated for title and abstract. At this step, 2,622 did not meet the eligibility criteria and were excluded. The remaining 43 studies were selected for full-text analysis. Of these, 31 studies were excluded as follow: co-treatment studies in which adverse events were not directly attributable to the epigenetic agents (18 papers); duplicates (4 studies); case report (9 studies). Finally, 12 studies were included in the final analysis. The selection process and study flow diagram are depicted in Figure 1.

### Quality Assessment

The included phase I and phase I/II trials showed overall adequate methodological quality. All studies clearly stated their aims and used endpoints appropriate to the objectives, with prospective data collection consistently performed. Scores were lower for unbiased endpoint assessment and prospective sample size calculation, reflecting common limitations of early-phase, single-arm trials. Total quality scores ranged from 9 to 12 out of a maximum of 16, indicating satisfactory methodological rigor for this trial phase. Overall, the studies had strong scores in defining objectives and endpoints, while limitations were expected for blinding, sample size calculation, and absence of control groups inherent to early-phase designs. The overall methodological quality of the 12 studies according to the MINORS scores is displayed in Table 1.

**Table 1:** Methodological quality assessment of the included studies using MINORS score (maximum possible score: 16).

			M. Mongje et al. 2023 [18]	*P*.J. Wood et al.2018 [19]	J. Goldberg et al. 2020 [20]	S. Mueller et al. 2023 [21]	C. M. van Tilburg et al. 2019 [22]	M. Fouladi et al.2010 [23]	J.M. Su et al.2022 [24]	J.E.A. Wolff et al. 2008 [25]	J.M. Su, et al. 2011 [26]	M. Fouladi et al. 2006 [27]	A. Bukowinski et al. 2021 [28]	A.*P*. Zorzi et al. 2013 [29]
	ITEMS													
	1	Clearly Stated Aim	2	2	2	2	2	2	2	2	2	2	2	2
	2	Inclusion of consecutive patients	1	1	2	1	2	1	2	1	2	2	2	1
	3	Prospective Collection of Data	2	2	2	2	2	2	2	2	2	2	1	2
	4	End Points Appropriate to Study Aim	2	2	2	2	2	2	2	2	2	2	2	2
	5	Unbiased Assessment of Study End Point	1	1	1	1	1	1	1	1	1	1	1	1
	6	Follow-Up Period Appropriate to Study Aim	1	1	1	1	1	1	1	2	1	0	1	1
	7	Less than 5% Lost to Follow Up	1	1	1	1	2	1	1	1	1	1	1	2
	8	Prospective Calculation of the Study Size	0	0	0	0	0	0	0	0	0	0	0	0
COMPARATIVE STUDIES	9	Adequate Control Group	n.a.	n.a.	n.a.	n.a.	n.a.	n.a.	n.a.	n.a.	n.a.	n.a.	n.a.	n.a.
	10	Contemporary Groups	n.a.	n.a.	n.a.	n.a.	n.a.	n.a.	n.a.	n.a.	n.a.	n.a.	n.a.	n.a.
	11	Baseline Equivalence of Groups	n.a.	n.a.	n.a.	n.a.	n.a.	n.a.	n.a.	n.a.	n.a.	n.a.	n.a.	n.a.
	12	Adequate Statistical Analysis	n.a.	n.a.	n.a.	n.a.	n.a.	n.a.	n.a.	n.a	n.a.	n.a.	n.a.	n.a.
		Total score	10	10	11	10	12	10	11	11	11	10	9	11

Items were scored 0–2; “n.a.” indicates not applicable for non-comparative studies

### Characteristics of the Included Studies

Of the 12 selected studies, 11 were single-arm non-randomized clinical trials clinical trials [18, 20–24, 26–29] and 1 was a retrospective study [25]. Phase I trials accounted for 9 studies [18–21, 23, 26–29], phase I/II trials for 2 studies [22, 24]. The selected studies evaluated six HDACi, including the pan-HDAC inhibitors panobinostat, vorinostat, and pracinostat, targeting class I, II, and IV HDACs [30–32]; valproic acid, which weakly inhibits class I HDACs [33]; and the class I-selective inhibitors depsipeptide and entinostat [34, 35]. All studies included in the analysis primarily aimed to evaluate the safety and toxicity profiles of the investigated agents, addressing at least two domains: MTD/RP2D, and AEs. A total of 415 subjects were included. All patients included in the selected studies presented refractory/resistant/progressive disease at the time of study inclusion, except for those diagnosed with glioma included also at diagnosis. About 12% of the total number of patients (*n* = 52) did not complete the trial they were enrolled in. Disease progression was the most frequent cause of treatment discontinuation across the included studies. It was observed in patients treated with panobinostat (*n* = 6) [19, 20] entinostat (*n* = 7) [28], vorinostat (*n* = 4) [23], valproic acid (*n* = 2) [26], and depsipeptide (*n* = 1) [27]. Toxicity-related discontinuations were less frequent but heterogeneous across studies, involving both hematologic and non-hematologic adverse events. Overall, a total of 9 patients discontinued treatment due to toxicity across these studies. During panobinostat treatment of leukemia cohort at 34 mg/m^2^, a total of 5 patients discontinued due to toxicity. These included 1 grade 5 invasive fungal infection (Aspergillus), 1 case of electrolyte abnormalities not responsive to supplementation, and 3 patients with gastrointestinal toxicity (2 with nausea/vomiting leading to early discontinuation and 1 with severe nausea, vomiting, and diarrhea causing dehydration) [20]. For vorinostat studies, 2 patients discontinued, one due to a serious adverse event at high dose of vorinostat [22], and one due to thrombocytopenia after a single dose in the dose escalation study [23]. In the study by Su et al., vorinostat administered at a dose of 230 mg/m² was discontinued in one patient due to neurological deterioration [24], while during depsipeptide treatment, 1 patient discontinued due to nausea/vomiting [27]. Other causes were mainly related to withdrawal, administrative decisions, or failure to start therapy. These included pre-treatment withdrawals or non-initiation, physician or family decision, infections, inability to reach drug levels, and patient refusal. Totally, data from 363 patients were considered. Overall, the median age was 11y (range: 2-21y), and the 54.2%±18.62 of subjects were male. Detailed characteristics of the included studies and demographic characteristics are summarized in Table 2.

**Table 2:** Characteristics of the included studies.

Study, y	Clinical trial ID	Design	Population, *N* (Evaluable patients at non-DLTs)	Median age (y) (range)	Male (%)	HDACi	Reference
Monje et al., 2023	NCT02717455	Phase I	DIPG / DMG, 13	7.1 (3.2–21.4)	46.0	Panobinostat	[18]
Wood et al., 2018	ACTRN12609000978268*	Phase I	Refractory solid/CNS tumors, 6	9 (3.0–13.0)	55.5	Panobinostat	[19]
Goldberg et al., 2020	NCT01321346	Phase I	Relapsed/refractory hematologic malignancies, 23	> 1 and < 21 years	Not specified	Panobinostat	[20]
Mueller et al., 2023	NCT03566199	Phase I	Newly diagnosed DIPG, 7	8 (5–21)	86.0	Panobinostat	[21]
van Tilburg et al., 2019	NCT01422499	Phase I/II	Relapsed solid tumors, lymphoma, leukemia, 50	11 (3.0–18.0)	56.0	Vorinostat	[22]
Fouladi et al., 2010	NCT00217412	Phase I	Refractory solid/CNS tumors, 42	11 (2.6–22.0)	63.5	Vorinostat	[23]
Jack M Su et al., 2022	NCT01189266 ACNS0927	Phase I/II	Newly diagnosed DIPG, 66	7.1 (3.3–19.4)	46.0	Vorinostat	[24]
Wolff et al., 2008	N.A.	Observational	HGG, 44	11.9 (3.6–17.5)	56.8	Valproic acid	[25]
Su et al., 2011	N.A.	Phase I	Refractory solid/CNS tumors, 16	13.5 (3.0–21.0)	38.4	Valproic acid	[26]
Fouladi et al., 2006	NCT00019318	Phase I	Refractory solid tumors, 23	13 (2.0–21.0)	47.8	Depsipeptide	[27]
Bukowinski et al., 2021	NCT02780804	Phase I	Recurrent/refractory solid/CNS tumors, 13	14 (6.0–20.0)	48.0	Entinostat	[28]
Zorzi et al., 2013	NCT01184274	Phase I	Refractory solid tumors, 12	≥ 3 and ≤ 18	66.6	Pracinostat	[29]

*DIPG* Diffuse Intrinsic Pontine Glioma, *DMG* Diffuse Midline Glioma, *CNS* Central Nervous System, *HGG* High-Grade Glioma, *DLT* Dose Limiting Toxicites

*Australian New Zealand Clinical Trials Registry (ANZCTR)

### Tumors Distribution

Four studies focused on gliomas [18, 21, 24, 25], including Diffuse Intrinsic Pontine Glioma (DIPG), Diffuse Midline Glioma (DMG), High-Grade Glioma (HGG), Glioblastoma (GBM), Anaplastic Astrocytoma (AA) (*n* = 183/415, 44%). Only one study involved hematologic malignancies, including acute myeloid leukemia (AML), acute lymphoblastic leukemia (ALL), Hodgkin lymphoma (HL), and non-Hodgkin lymphoma (NHL) (*n* = 27/415, 6.5%)[20]. The remaining 7 studies included heterogeneous populations comprising mixed solid/non-solid/CNS tumors (*n* = 205/415, 49.4%) [19, 22, 23, 26–29]. Among studies enrolling patients with multiple solid tumors Central Nervous System (CNS) accounted for the largest proportion of cases (44.4%), followed by connective tissue, including bone and soft tissue sarcomas (34.7%) and extracranial solid (13.8%), while leukemia and lymphoma accounted for 3% and less than 1% tumor respectively. Frequencies of disease/population of the 7 mixed population studies are reported in supplementary file 2.

## Toxicities

Toxicities across studies were graded according to the NCI Common Terminology Criteria (CTCAE) scale. We evaluated toxicities at MTD/RP2D in AEs dose-finding studies. Four dose-escalation studies involving vorinostat, valproic acid, and aqueous panobinostat reported the overall incidence of AEs regardless of dosage and were analyzed separately [21–23, 26]. AEs per class are reported in Table 3. The overall frequencies of grade 1–5 is reported in Table 4. Detailed AEs are reported in supplementary file 3.

**Table 3:** Treatment-related adverse events of HDACi.

HDACi; Population; *N*; Dose; Route of administration	Hematological AEs	GI AEs	Metabolic / Biochemical AEs	Cardiac AEs	Neurological AEs	Respiratory AEs	Systemic AEs
Panobinostat; DIPG; *N* = 13; 10 mg/m²/dose; PO [18]	Thrombocytopenia 77.0% (7.6% G3); ↓WBC 23.0% (1 pt G4); Neutropenia 15.3% (1 pt G4); Lymphopenia 53.8% (23.0% G3)	↑ ALT 61.5% (7.5% G3); Vomiting 11 Diarrhea 6.0%; Nausea 14.0%	Hypokalemia 15.4%; Hypocalcemia 15.4%; Hypoalbuminemia 46.0%	–	Encephalopaty syndrome 7.6%	–	Fatigue 38.4%; Sepsis 7.6% (G4)
Panobinostat; Mixed solid/CNS; *N* = 6; 15 mg/m²/dose; IV [19]	Thrombocytopenia 100% (33.3% G3); Anemia 31.0%; ↓WBC 32%; Neutropenia 67.0%; Lymphopenia 16.0%	↑ ALT/AST 16.6%; Vomiting 16.6%; Constipation 16.6%	Hypokalemia 39.0%; Hypocalcemia 16.6%; hypercreatininemia 16.6%	QTc prolongation 16.6%	Hydrocephalus; Gait abnormality 16.6% (G3)	Dyspnea 16.6% (G3); Hypoxia 16.6% (G3); Apnea 16.6% (G4)	Fatigue 33.0%
Panobinostat; Hematological malignancies; *N* = 23; 34 mg/m²/day; PO [20]	Thrombocytopenia 56.5%; Anemia 69.6%; ↓WBC 47.8%; Neutropenia 39.0%; Lymphopenia 39.0%; Febrile neutropenia 52.0%	Diarrea 17.4%; hypertrigliceremia 21.7%	Hypophosphatemia 17.4%; Hyperglycemia 39.0%	–	–	Hypoxia 13.0%	–
Panobinostat; DIPG; *N* = 7; 30–90 µM; CED; [21]	Neutropenia 14.3%; Lymphopenia 28.6%; ↓WBC 14.3%	↑ ALT 14.3%	Hyperglycemia 14.8%; Hypomagnesemia 14.8%	QTc prolongation 28.6%	Dysgeusia 14.2%	–	Fatigue 14.3%
Vorinostat; Solid tumors; *N* = 50; 130–580 mg/m²; PO [22]	Thrombocytopenia 74.0% (32.0% G4); Anemia 20.0%; ↓WBC 18.0%	Nausea 30.0%; Vomiting 20.0%; ↑ ALT 8.0%; Constipation 8.0%	Hypermagnesemia 6.0%	–	–	–	Fatigue 32.0%; Weight loss 16.0%; Headache 12.0%
Vorinostat; *Arm A* Solid tumors; *N* = 24; Starting dose 180 mg/m²; PO [23]	Thrombocytopenia 44.0%; Anemia 26.4%; ↓WBC 23.5% (2.9% G4); Neutropenia 29.0%	Nausea 20.5%; Vomiting 23.5%; Diarrhea 17.6%; AST/ALT ↑ 11.7%	Hypocalcemia 14.7%	–	–	–	–
Vorinostat; *Arm B* Hematologic malignancies; *N* = 6; 230 mg/m²; PO [23]	–	Increase ALT 7.8%; Nausea 33.0%; Vomiting 33.0%	Cretininemia 16.6%	–	–	–	Fatigue 41.0%; Anorexia 33.0%
Vorinostat; *Arm D* Solid tumors; *N* = 12; 50 mg/mL; PO solution [23]	↓WBC 41.0%; Lymphopenia 33.0%; Thrombocytopenia 25.0%; Neutropenia 25.0%; Anemia 16.6%	–	Hypophosphatemia 8.3%	–	–	–	Fatigue 8.3%
Vorinostat; *Arm C* DIPG; *N* = 66; 230 mg/m²; PO [24]	Thrombocytopenia 13.6% (4.5% G4); Neutropenia 7.5% (all G4)	–	Hypermagnesemia 3.0%	–	Intracranial hemorrage 1.5% (G4)		Depressed consciusness 1.5%
Valproic acid; HGG; *N* = 44; 10 mg/kg/day; PO [25]	Thrombocytopenia 2.2% (G1–2); Anemia 2.2% (G1–2)	–	–	–	difficulties in concentrating; Increased sensitivity to light 2.0%		Somnolence 7.0%
Valproic acid; Mixed solid/CNS; *N* = 16; Starting dose 25 mg/kg/day; PO [26]	Thrombocytopenia 68.7% (6.2% G4); Anemia 56.2% (6.2% G4); ↓WBC 50.0%; Neutropenia 25% (6.2% G4); Lymphopenia 56.2% (6.2% G4)	Nausea 12.5%; Vomiting 12.5%	Hypoalbuminemia 18.7%	–	–	–	Fatigue 31.3%; somnolence 12.5%
Depsipeptide; Solid tumors, *N* = 23; 17 mg/m²; IV [27]	Thrombocytopenia 13.0% (4.3% G4); ↓WBC 17.0% (4.3% G4); Neutropenia 26.0% (8.6% G4); Lymphopenia 13.0% (4.3% G4)	Nausea 4.3%; AST/ALT ↑ 8.0–13.0%	Hypophosphatemia 4.3%; Hypocalcemia 4.3%	QTc prolongation 4.3%; Sick sinus syndrpme 4.3%	–	–	Fatigue 8.6%; Anorexia 17.3 (G3); Weight loss 4.3%
Entinostat; Mixed solid/CNS; *N* = 13; 4 mg/m²; PO [28]	Thrombocytopenia 69.2%; Anemia 7.6%; ↓WBC 46.0% (7.6% G3); Neutropenia 38.4% (23% G3)	Increased ALT/AST 23.0–30.0%; Nausea 15.4%; Vomiting 23.0%; Diarrhea, Consitpation 15.4%	Hypophosphatemia 23.0%; Hypokalemia 23.0%; hypermanesemia 23.0; others < 10%	–	–	–	–
Pracinostat; Solid tumors; *N* = 12; 45 mg/m²; PO [29]	Thrombocytopenia 33.3% (8.3% G3); Anemia 33.3% (G2); Neutropenia 25.0% (16.6% G3)	Nausea 8.3%; Vomiting 8.3%; Increased ALT/AST 8.3% (G3); Consipation 16.6%	Creatininemia 16.6%; Hypocalcemia 8.3%; Hypokalemia 8.3%	–	–	Dyspnea 8.3%; Epistaxis 8.3%; Cough 8.3%	Weight loss 8.3%

AEs are grouped by organ system and reported as frequencies; toxicity grade is specified when ≥ 3

*G* Grade (according to CTCAE criteria), *pt* patient, *WBC* White Blood Cells, *ALT* Alanine Aminotransferase, *AST* Aspartate Aminotransferase, *QTc* corrected QT interval, *AEs* Adverse Events, *GI* Gastrointestinal, *CED* convention enhanced delivery, *PO* Per Os, *IV* Intravenous, *RP2D* Recommended Phase 2 Dose, *DIPG* Diffuse Intrinsic Pontine Glioma, *HGG* High-Grade Glioma, *CNS* Central Nervous System

Blank cells indicate not reported events

**Table 4:** AEs by grade (Grade 1–5, CTCAE).

Treatment	*N*°	AEs total (*n*)	G1- G2 (*n*/%)	G3 (*n*/%)	G4 (*n*/%)	DOI
Panobinostat	6	84	64/76	17/20	3/3.5	[19]
Panobinostat	7	14	13/93	1/7	0	[21]
Panobinostat	23	95	n.a.	94/99	1/1	[20]
Panobinostat	13	65	47/72	12/18	6/9	[18]
Vorinostat	24	130	113/87	16/12	1/1	[23]
Vorinostat	6	26	18/69	8/31	0	[23]
Vorinostat	12	23	16/69	6/26	1/4.3	[23]
Vorinostat	50	171	107/62	48/28	16/9	[22]
Vorinostat /mainteinance	66	25	3/12	13/52	9/36	[24]
Valproic acid	26	52	43/83	5/10	4/7	[26]
Depsipeptide	23	40	22/55	13/32	5/12	[27]
Entinostat/cycle 1	13	61	58/94	4/6	0	[28]
Entinostat/cycle 2	13	39	37/95	2/5	0	[28]
Pracinostat	12	37	32/86	5/13	0	[29]

Percentages refer to total AEs

*N*° patients, *n.a.* not available

### Hematologic Toxicity

Across AEs dose-stratified analyses of adverse events hematologic toxicities were the most frequently reported events with HDACi and showed clear dose dependence. Thrombocytopenia was consistently the predominant toxicity. The highest incidence was observed with panobinostat regardless of dose, reported in 56.50 up to 100% of patients, with grade ≥ 3 events occurring in more than on one-third of cases (33.3%) [18–21]. Similarly, elevated rates of thrombocytopenia were reported with entinostat (69.2%) and pracinostat (33.3%) [28, 29]. A lower incidence was observed with vorinostat oral suspension (25.0%), predominantly grade 1–2 events [23], as well as in the maintenance setting (13.6%); however, grade 4 events still occurred in 4.5% of patients [24]. Valproic acid showed variable rates ranging from 2.0% in retrospective analyses [25] to 68.7% in phase I trials (including 6.2% grade 4 cases) [26]. Neutropenia was the second most common toxicity. For panobinostat, a dose-dependent pattern was observed, occurring in 15.3% of patients at 10 mg/m² (DIPG cohort), 39.0% at 34 mg/m² (leukemia cohort), and 67.0% at 15 mg/m² (mixed solid tumor cohort). Grade ≥ 3 febrile neutropenia was reported in 52.0% of patients in the panobinostat-treated leukemia cohort [18–20]. Entinostat was associated with neutropenia in 38.4% of patients at a dose of 4 mg/m², including 15.4% grade 3 events (*n* = 2) [28]. Depsipeptide, administered at 17 mg/m², caused neutropenia in 26.0% of patients, including 8.6% grade 4 events (*n* = 2) [27]. Similarly, neutropenia occurred in 25.0% of patients treated with pracinostat at 45 mg/m², with 16.6% experiencing grade 3 toxicity (*n* = 2) [29]. A lower incidence was reported in the maintenance setting with vorinostat, mainly associated with grade 1–2 neutropenia observed in 25.0% of patients treated at 50 mg/m² [23], whereas the higher dose of 230 mg/m² was associated with 7.5% grade 4 neutropenia (*n* = 5) [24]. Lymphopenia was a relatively common but variable hematologic toxicity observed across different HDACi and disease settings. In contrast to neutropenia, lymphopenia did not exhibit a clear dose-dependent pattern in patients treated with panobinostat, occurring in 16.0% of patients with mixed solid tumours treated at 15 mg/m², 39.0% of patients with leukemia treated at 34 mg/m², and 53.8% of patients with DIPG treated at 10 mg/m² [18–20]. Lymphopenia was less frequent in patients treated with vorinostat; only 33.0% of those receiving maintenance therapy at a dose of 50 mg/mL experienced grade 1–2 lymphopenia [24]. Additionally, 13.0% of patients treated with depsipeptide at a dose of 17 mg/m² developed lymphopenia, including 4.3% (*n* = 1) with grade 4 severity [27]. Anemia occurred in 31.0% and 69.5% of patients receiving panobinostat at doses of 15 mg/m² and 34 mg/m², respectively [19, 20], and in 33.3% of patients treated with pracinostat at 45 mg/m² [29]; vorinostat, administered as maintenance therapy at 50 mg/ml, was associated with low-grade anemia in 16.6% of patients [23]. Decreased white blood cell (WBC) counts were less consistently reported and were predominantly grade 1–2 across all HDAC inhibitors, with isolated grade 4 events observed in patients treated with panobinostat at 10 mg/m² (*n* = 1) [18] and depsipeptide at 17 mg/m² (*n* = 1) [27]. Hematological toxicity remained the dominant adverse event category also in not dose-stratified AEs analyses, particularly at high doses, with thrombocytopenia representing the most frequent manifestation. The highest incidence was observed with high-dose vorinostat, affecting 74.0% of patients, including 32.0% with grade 4 toxicity [22]. This was followed by high-dose valproic acid, with an incidence of 68.7%, including 6.2% grade 4 cases (*n* = 1) [26]. Anemia occurred in 20.0% and 26.4% of vorinostat-treated patients [22, 23] and in 56.2% of high-dose valproic acid treatment, including 6.2% grade 4 cases (*n* = 1) [26]. Decreased WBC counts were reported in 18.0% [22] and 23.5% of vorinostat cohorts (up to 2.9% grade 4, *n* = 1) [23], in 50.0% patients treated with high-dose valproic acid, including 2.7% grade 4 cases (*n* = 1) [26], and in 14.3% of patients receiving panobinostat [21]. Neutropenia occurred in 29.0% of patients treated with vorinostat [23], compared with 14.3% of patients treated with panobinostat [21] and 25.0% of patients receiving high-dose valproic acid, including 6.2% with grade 4 toxicity (*n* = 1) [26]. Lymphopenia was less frequently reported, occurring in 28.6% of subjects treated with panobinostat [21] and and in 56.2% of patients treated with high-dose valproic acid, including 2.7% with Grade 4 events (*n* = 1) [26]. Overall, both dose-stratified and non–dose-stratified analyses consistently identify hematologic toxicity, particularly thrombocytopenia, as the principal adverse effect of HDACi. Incidence and severity increased with higher doses treatments, particularly with panobinostat and high-dose vorinostat and valproic acid, whereas maintenance regimens showed comparatively lower or more variable rates of thrombocytopenia, neutropenia, anemia, lymphopenia, and decreased WBC counts.

### Gastrointestinal Toxicity

Gastrointestinal toxicities were generally mild (grade 1–2) across all HDACi. In dose-stratified studies, nausea and vomiting were the most frequently reported adverse events, each occurring in 33.0% of patients with leukemia treated with vorinostat at a dose of 230 mg/m^2^ [23]. Lower and comparable rates were observed with entinostat (15.0%) and panobinostat (14.0%) [18–20, 28]. Diarrhea was reported less frequently, with incidence rates ranging from 6.0% in patients treated with panobinostat at 10 mg/m² to 17.0% in those treated at 34 mg/m² [18, 20] and approximately 15.0% with entinostat [28], while constipation was sporadically observed, affecting 6.0–16.0% of patients. Hypertriglyceridemia was reported in 21.7% of leukemia patients treated with 230 mg/m^2^ of panobinostat [20]. Hepatic toxicity emerged as the most relevant dose-dependent adverse event. Elevation in AST and ALT occurred most frequently with panobinostat across all dose levels (up to 61.5%, including 7.5% grade-3, *n* = 1) [18, 19] and entinostat (AST: 23.0%; ALT: 30.0%)[28], followed by depsipeptide (AST: 8.0%; ALT: 13.0%)[27]. Grade 3 transaminasitis were also observed with pracinostat (8.3%, *n* = 1) [29]. In non–dose-stratified analyses, gastrointestinal events remained predominantly mild and were reported at generally lower and more homogeneous incidence rates. Vorinostat was associated with nausea (from 20.0% up to 30.0%) and vomiting (around 20.0%) [22, 23], whereas valproic acid induced only mild nausea and vomiting (12.5%) [26]. Diarrhea was not frequently reported, and constipation was uncommon. Transaminase elevations were observed in a minority of patients treated with vorinostat and panobinostat (approximately 8.0–14.0%) [21–23]. Overall, nausea and vomiting represent the most common gastrointestinal adverse events across HDACi, whereas AST and ALT elevations represent the principal dose-related toxicity, particularly in patients treated with panobinostat and entinostat.

### Metabolic and Electrolyte Toxicity

Metabolic adverse events were generally rare, mild, and sporadic across studies. In dose-stratified trials, electrolyte abnormalities were more frequently observed with panobinostat, entinostat, and pracinostat, particularly at higher doses. Hypokalemia was the most common metabolic event, occurring in 15.0% up to 39.0% of patients treated with panobinostat across dose levels [18–20], and in 23.0% of those receiving entinostat [28]. Pracinostat was mainly associated with elevated serum creatinine levels (16.6%, *n* = 2)[29], while depsipeptide showed a very low incidence of mild metabolic toxicity reported in up to 4.3% (*n* = 1) of the patients [27]. In contrast, vorinostat and valproic acid were only rarely associated to metabolic disturbances in dose-stratified analyses. Non–dose-stratified studies confirmed a similar pattern, although with slightly lower and more heterogeneous incidences. Panobinostat via convection-enhanced delivery (CED) was associated with hyperglycemia and hypomagnesemia in 14.8% (*n* = 1) of patients [21], while vorinostat at starting dose of 180 mg/m^2^ induced hypocalcemia in 14.7% subjects [23]. Hypoalbuminemia was reported in 18.7% of patients treated with high-dose valproic acid [26]. Overall, dose-stratified data suggest a dose-dependent increase in metabolic and electrolyte abnormalities, particularly with panobinostat and entinostat, whereas non-dose-stratified studies provide slightly more homogeneous incidence.

### Systemic

Severe systemic adverse events were uncommon. A notable exception was panobinostat, for which grade 4 sepsis was reported in 7.6% (*n* = 1) of patients treated at a dose of 10 mg/m^2^ [18]. Fatigue consistently emerged as the most prevalent systemic toxicity across HDACi. This was particularly frequent during treatment with panobinostat at doses of 10 and 15 mg/m², with incidence rates of 33.0% and 38.4%, respectively [18, 19] and less common in non–dose-stratified cohorts (14.3%) [21]. Vorinostat was also associated with dose-dependent rates of fatigue, occurring in 41.0% of leukemia patients treated at 230 mg/m² and in 8.3% of patients affected by solid tumors treated at 50 mg/m² [23]; in a non–dose-stratified cohort, fatigue was reported in 32.0% of patients [22]. In contrast, fatigue was less frequent with depsipeptide (8.6%, *n* = 2)[27]. Other constitutional symptoms were less consistently observed. Somnolence was observed exclusively in patients treated with valproic acid, occurring more frequently at higher doses (12.5% at a starting dose of 25 mg/kg) and less commonly at the lower dose of 10 mg/kg (7.0%) [25, 26]. Anorexia occurred at moderate rates, particularly with 230mg/m^2^ of vorinostat (up to 33.0% in hematologic patients)[23] and depsipeptide (17.3%, including two grade 3 cases) [27], while it was not a prominent toxicity with panobinostat or other HDACi. Weight loss was less frequent overall, mainly described with vorinostat in dose-escalation studies (16.0%, grade 1–3) [22], and only sporadically reported with depsipeptide and pracinostat. In summary, fatigue represents the dominant systemic adverse event across studies, whereas other constitutional symptoms such as anorexia, weight loss, and somnolence were less frequent and more treatment-specific.

### Respiratory

Respiratory adverse events were generally infrequent but included clinically relevant cases of dyspnea, hypoxia, and, rarely, apnea. Grade 2–3 dyspnea was reported in 16.6% of patients (*n* = 1) treated with panobinostat at a dose of 15 mg/m² [19] and in 8.3% of patients (*n* = 1) treated with pracinostat [29]. Hypoxia was reported in 13.0% of hematologic patients treated with panobinostat at a dose of 34 mg/m^2^ [20]. Severe respiratory events were rare. Grade 3 hypoxia occurred in 13.0% of patients in the leukemia cohort treated with panobinostat at 35 mg/m² [20], while a single case of grade 4 apnea (16.6%) was reported among patients receiving panobinostat at 15 mg/m² [19]. Other respiratory adverse events, such as cough, were rare and predominantly mild.

### Neurological

Neurological AEs were rare and generally low grade. The most clinically relevant neurological event was an intracranial hemorrhage associated with maintenance vorinostat at a dose of 230 mg/m², reported in 1.5% of patients (*n* = 1; grade 4) [24]. Abnormal gait and hydrocephalus were observed in 16.6% of patients treated with panobinostat at 15 mg/m² [19]. Other mild neurological events, including, difficulty concentrating, dysgeusia, and increased sensitivity to light, were infrequently reported across studies of panobinostat via CED and valproic acid at low-dose.

### Cardiac

Cardiac adverse events were rare and were reported in studies of panobinostat and depsipeptide. QTc prolongation was observed at grade 1–2 severity in 16.6% (*n* = 1) of patients treated with 15mg/m^2^ panobinostat [19], in 28.6% (*n* = 2) of patients receiving a aqueous panobinostat [21], and in 4.3% (*n* = 1) of patients treated with depsipeptide at 17 mg/m^2^ [27]. Other grade 1–2 cardiac events included arrhythmia (16.6%, *n* = 1, panobinostat) [19] and sick sinus syndrome (4.3%, depsipeptide) [27].

### Other AEs

Cutaneous adverse events were reported exclusively in patients treated with vorinostat at high doses, including alopecia (18.0%, *n* = 9) and dry skin (6.0%, *n* = 3) [22].

## Discussion

Across pediatric studies of HDACi, adverse events are predominantly mild to moderate in severity. In most datasets, grade 1–2 toxicities represent the vast majority of events, often accounting for more than 70–90% of reported adverse effects, whereas grade 3 events are less frequent, and grade 4 events are uncommon or absent. Although variability exists among individual agents and studies, the overall safety pattern suggests that most toxicities remain within lower grades and are generally manageable. Higher toxicity profiles are observed with pan-HDACi, yet these are typically considered acceptable within early-phase trial settings, supporting the rationale for exploring combination strategies to enhance therapeutic efficacy. From a mechanistic standpoint, pan-HDACi such as vorinostat and panobinostat induce broad acetylation of histone and non-histone proteins, resulting in widespread transcriptional activation, impaired DNA repair, and increased proteotoxic stress [31, 36, 37]. Consistent with this wide biological activity, they are associated with the highest toxicity burden, particularly hematologic toxicity. Across studies, panobinostat and vorinostat show the most frequent and severe cytopenias, including mainly thrombocytopenia and neutropenia, which are clearly dose-dependent and often define the MTD. Pracinostat, although also a pan-HDAC inhibitor, displays an intermediate hematologic toxicity profile, closer to that of class I–selective agents such as entinostat. Notably, both entinostat and pracinostat show fewer consistent or recurrent dose-limiting toxicities across cohorts. Valproic acid, a weak class I HDAC inhibitor, produces only modest increases in histone acetylation and limited direct cytotoxicity [33]. Accordingly, toxicity is generally milder at low doses, although higher exposures can still lead to hematologic suppression and occasional metabolic or respiratory disturbances, including electrolyte imbalance. Overall, hematologic toxicity consistently emerges as the hallmark adverse effect of systemic HDAC inhibition, especially for panobinostat and vorinostat, demonstrating a clear dose–response relationship and frequently representing the dose-limiting factor that determines RP2D. Thrombocytopenia is the most prominent event, followed by neutropenia, whereas anemia is less frequently dose-limiting. Non-hematologic toxicities are generally less severe and include gastrointestinal effects (nausea, vomiting, diarrhea), hepatotoxicity (transaminase elevation), metabolic abnormalities (hypokalemia, hypertriglyceridemia), and fatigue, the latter being the most consistently reported systemic symptom. Gastrointestinal toxicity is common but usually low-grade, although grade ≥ 3 events have been reported with pracinostat and entinostat. Hepatotoxicity, primarily transaminase elevation, appears dose-related and is more evident with panobinostat and entinostat. Cardiac toxicity (notably QTc prolongation), as well as neurologic and respiratory events, remain rare but clinically relevant. Patterns of DLTs further reflect these differences: hematologic DLTs predominate with panobinostat and vorinostat, while additional DLTs include cardiac events (panobinostat, depsipeptide), neurologic toxicity (e.g., MTX110, aqueous panobinostat formulations), metabolic disturbances such as hypertriglyceridemia (panobinostat), and electrolyte or respiratory complications (valproic acid). Overall, the integrated evidence supports a clear relationship between HDAC isoform breadth, pharmacologic potency, and toxicity burden, with broader inhibitors showing greater toxicity but also stronger antitumor activity, thereby providing a strong rationale for optimized dosing and rational combination strategies to improve the therapeutic index. In this context, comparison with adult data is informative. Although direct comparison between pediatric and adult populations is limited by differences in tumor types, dosing schedules, prior therapies and trial design, the available adult literature suggests a broadly similar toxicity spectrum for HDAC inhibitors, dominated by hematologic toxicity, gastrointestinal symptoms, fatigue and QTc prolongation. However, grade 3 or higher events appear more frequent in adult studies, particularly at full therapeutic doses and in combination regimens [12, 38]. In pediatric studies, severe toxicities were less frequent, reporting mainly grade 1–2 adverse events, with severe toxicities being relatively uncommon, although hematologic toxicity remains the most consistently observed domain. These differences likely reflect lower MTD in children, developmental pharmacokinetic and pharmacodynamic variability, and the early-phase nature of pediatric trials, which limits cumulative exposure. Overall, while toxicity patterns are qualitatively comparable between adults and children, long-term safety data remain limited, particularly in relation to developmental processes, underscoring the need for extended follow-up in pediatric cohorts [39]. In addition, potential long-term risks of HDAC inhibition, including effects on reproductive function and the theoretical risk of secondary malignancies, should be considered [40]. Potential long-term risks, including effects on fertility, gonadal maturation, neurodevelopment and the theoretical risk of secondary malignancies, remain insufficiently characterized in children. HDACs participate in germ-cell development and tissue maturation, raising particular concern in prepubertal patients. Available clinical studies have not demonstrated a clear signal for secondary malignancies, but follow-up remains too limited to exclude rare or delayed effects. Long-term prospective surveillance should therefore be incorporated into future pediatric HDACi trials. Therefore, these risks remain theoretical in this setting and cannot be adequately quantified from available pediatric studies. However, the evaluation of toxicities induced by epigenetic agents in pediatric Phase I clinical trials faces significant structural and biological hurdles that challenge the reliability of current safety data. First, a primary limitation is the delayed manifestation of epigenetic toxicity. Unlike conventional cytotoxic chemotherapy, which typically induces acute side effects, epigenetic modifiers work through gradual cellular reprogramming. Consequently, DLTs or developmental impairments may emerge months or years after the initial treatment, far exceeding the standard 21-to-28-day observation window of Phase I trials [41, 42]. Furthermore, the reliance on Body Surface Area (BSA)-based dosing (mg/m^2^) introduces substantial variability. In pediatric cohorts ranging from infants to adolescents, BSA-based calculations often fail to account for the age-dependent maturation of metabolic enzymes and organ function. This discrepancy means that the same dose can lead to vastly different systemic exposures and unpredictable toxicity profiles across different age groups. Because of this extreme pharmacokinetic heterogeneity, toxicity data derived from small pediatric cohorts cannot be considered definitive or universally applicable [43]. These factors, combined with the lack of validated “epigenomic” safety biomarkers, suggest that current Phase I safety reports may offer only an incomplete and potentially misleading “snapshot” of the true long-term risk profile in children. Furthermore, the interpretation of pooled toxicity data is limited by substantial heterogeneity across studies. Most included trials were phase I or phase I/II dose-escalation studies designed primarily for dose finding and MTD determination rather than for comparative safety assessment. These early-phase designs involve small sample sizes and cohort-based escalation schemas, introducing intrinsic variability in toxicity reporting. Additional heterogeneity arises from differences in formulation and dose intensity relative to adult regimens. Most studies employed fixed BSA dosing for panobinostat, vorinostat, entinostat, and pracinostat whereas valproic acid trials relied on target serum concentration–guided dosing [25, 26], reflecting a pharmacokinetically driven approach. Such differences in exposure targeting complicate cross-study comparisons. Route of administration further influences toxicity profiles. While most pediatric studies used oral formulations of panobinostat and intravenous administration has been explored in selected contexts, convection-enhanced delivery (MTX110) has been employed in DIPG to minimize systemic exposure. These differences likely contribute to variation in hematologic toxicity rates. Moreover, pediatric MTDs are frequently lower than those used in adult populations [36–40], limiting direct comparability and potentially underestimating toxicity relative to adult experience. A major strength of this analysis is the relative consistency of the median age across studies. Despite a broad age range (1–21 years), most enrolled patients were preadolescent, enhancing the comparability of toxicity assessments across trials. Such homogeneity likely minimizes variability in drug pharmacokinetics and reduces potential bias that might arise from cohorts with more heterogeneous age distributions. Taken together, heterogeneity in study phase, dose-escalation design, exposure targeting (fixed mg/m² versus serum-guided), formulation (oral, intravenous, or local delivery), and relative dose intensity represents a structural source of bias that constrains robust cross-trial comparisons of adverse event profiles.

## Conclusion

Overall, pediatric data indicate that HDAC inhibitors have a largely predictable, dose-dependent toxicity profile, with hematologic toxicity as the principal safety signal, especially with pan-HDAC inhibitors at higher doses. Non-hematologic adverse events are generally infrequent and manageable, supporting their clinical use in pediatric oncology when appropriate monitoring and dosing strategies are applied. These findings support the need for careful hematologic, hepatic, metabolic and cardiac monitoring in pediatric patients receiving HDAC inhibitors. At the same time, they indicate that the clinical use of this drug class should rely on optimized dosing schedules, appropriate patient selection and rational combination strategies aimed at improving the therapeutic index. The current clinical promise of HDAC inhibitors as therapeutic options in pediatric oncology is supported by emerging evidence of biological activity in epigenetically driven tumors, while the summarized AE profiles define a relatively narrow but manageable therapeutic window that directly informs dose optimization and the design of combination strategies [44–46]. However, the interpretation of these findings remains inherently limited. Epigenetic therapies act through slow cellular reprogramming, raising the possibility of delayed toxicities that may not emerge within standard short Phase I observation windows. In parallel, BSA-based dosing introduces meaningful pharmacokinetic variability across developmental ages, and the absence of validated epigenomic biomarkers further restricts early detection of subtle or evolving toxicity. As a result, current Phase I evidence likely provides only a partial view of the true long-term safety profile of HDACi in children. More refined, biomarker-informed and longitudinal studies are needed to better capture delayed effects and optimize safe clinical integration.

## Supplementary Information

Supplementary Material 1.

Supplementary Material 2.

Supplementary Material 3.

## Data Availability

No datasets were generated or analysed during the current study.
