# Supplementary material for: Mapping the Safety Profile of Histone Deacetylase Inhibitors in Children with Cancer
Source: Clin Epigenetics. 2026 Jul 7;18:175. doi: 10.1186/s13148-026-02192-z (PMC13625369; doi:10.1186/s13148-026-02192-z)
Supplement: Supplementary file 2 [file 13148_2026_2192_MOESM2_ESM.docx]

| **Category** | **Diagnosis** | **N** | **Frequency (%)** |
| --- | --- | --- | --- |
| Central CNS | High-grade / Malignant glioma (incl. Glioblastoma) | 35 | 14.46 |
| Central CNS | Medulloblastoma | 28 | 11.57 |
| Central CNS | Ependymoma | 18 | 7.43 |
| Central CNS | Glioma (low grade + mixed, excluding GBM) | 6 | 2.47 |
| Central CNS | Atypical teratoid/rhabdoid tumor (ATRT) | 4 | 1.65 |
| Central CNS | Pineoblastoma | 3 | 1.23 |
| Central CNS | Brainstem glioma | 3 | 1.23 |
| Central CNS | Diffuse intrinsic pontine glioma (DIPG) | 1 | 0.41 |
| Central CNS | Anaplastic oligoastrocytoma | 1 | 0.41 |
| Central CNS | Desmoplastic infantile ganglioglioma | 1 | 0.41 |
| Peripheral CNS | Primitive neuroectodermal tumor (PNET) | 8 | 3.3 |
| Soft tissue /bone sarcoma | Ewing sarcoma / Ewing family tumors | 20 | 8.26 |
| Soft tissue /bone sarcoma | Rhabdomyosarcoma (all subtypes) | 18 | 7.43 |
| Soft tissue / bone sarcoma | Osteosarcoma | 15 | 6.19 |
| Soft tissue /bone sarcoma | Soft tissue sarcoma (NOS) | 9 | 3.71 |
| Soft tissue /bone sarcoma | Synovial sarcoma | 8 | 3.3 |
| Soft tissue /bone sarcoma | Desmoplastic small round cell tumor (DSRCT) | 3 | 1.23 |
| Soft tissue /bone sarcoma | Spindle cell sarcoma | 1 | 0.41 |
| Soft tissue /bone sarcoma | Mesenchymal chondrosarcoma | 1 | 0.41 |
| Soft tissue /bone sarcoma | Alveolar soft part sarcoma | 1 | 0.41 |
| Soft tissue /bone sarcoma | Triton tumor | 1 | 0.41 |
| Soft tissue /bone sarcoma | Sarcoma, NOS | 1 | 0.41 |
| Extracranial/solid | Neuroblastoma | 14 | 5.78 |
| Extracranial/solid | Wilms tumor / Nephroblastoma | 8 | 3.3 |
| Extracranial/solid | Hepatocellular carcinoma, fibrolamellar | 2 | 0.82 |
| Extracranial/solid | Endodermal sinus (yolk sac) tumor | 2 | 0.82 |
| Extracranial/solid | Hepatoblastoma | 1 | 0.41 |
| Extracranial/solid | Adrenocortical carcinoma | 1 | 0.41 |
| Extracranial/solid | Paraganglioma | 1 | 0.41 |
| Extracranial/solid | SETTLE tumor | 1 | 0.41 |
| Extracranial/solid | Malignant germ cell tumor | 1 | 0.41 |
| Hematologic | Acute lymphoblastic leukemia (ALL) | 4 | 1.65 |
| Hematologic | Acute myeloid leukemia (AML incl. APL) | 3 | 1.23 |
| Hematologic | Lymphoma | 1 | 0.41 |
| — | **Total** | **225*** | **100.00** |

Supplementary table 2. Diseases distribution across the 7 solid/CNS tumors studies.

*Included the efficacy population (n=27) of the study of Cornelis M. van Tilburg, 2019.
